# *Plasmodium* infection, anaemia and mosquito net use among school children across different settings in Kenya

**DOI:** 10.1111/j.1365-3156.2012.03001.x

**Published:** 2012-05-11

**Authors:** Caroline W Gitonga, Tansy Edwards, Peris N Karanja, Abdisalan M Noor, Robert W Snow, Simon J Brooker

**Affiliations:** 1 Malaria Public Health & Epidemiology Group, Kenya Medical Research Institute-Wellcome Trust Research ProgrammeNairobi, Kenya; 2 Faculty of Infectious and Tropical Diseases, London School of Hygiene and Tropical MedicineLondon, UK; 3 MRC Tropical Epidemiology Group, London School of Hygiene and Tropical MedicineLondon, UK; 4 Centre for Tropical Medicine, Nuffield Department of Clinical Medicine, University of OxfordCCVTM, Oxford, UK

**Keywords:** *Plasmodium*, malaria, anaemia, insecticide-treated nets, risk factors, school children, Kenya

## Abstract

**Objective:**

To investigate risk factors, including reported net use, for *Plasmodium* infection and anaemia among school children and to explore variations in effects across different malaria ecologies occurring in Kenya.

**Methods:**

This study analysed data for 49 975 school children in 480 schools surveyed during a national school malaria survey, 2008–2010. Mixed effects logistic regression was used to investigate factors associated with *Plasmodium* infection and anaemia within different malaria transmission zones.

**Results:**

Insecticide-treated net (ITN) use was associated with reduction in the odds of *Plasmodium* infection in coastal and western highlands epidemic zones and among boys in the lakeside high transmission zone. Other risk factors for *Plasmodium* infection and for anaemia also varied by zone. *Plasmodium* infection was negatively associated with increasing socio-economic status in all transmission settings, except in the semi-arid north-east zone. *Plasmodium* infection was a risk factor for anaemia in lakeside high transmission, western highlands epidemic and central low-risk zones, whereas ITN use was only associated with lower levels of anaemia in coastal and central zones and among boys in the lakeside high transmission zone.

**Conclusions:**

The risk factors for *Plasmodium* infection and anaemia, including the protective associations with ITN use, vary according to malaria transmission settings in Kenya, and future efforts to control malaria and anaemia should take into account such heterogeneities among school children.

## Introduction

Insecticide-treated nets (ITNs), and more recently long lasting insecticide nets (LLINs), are a key tool in the control of malaria, with demonstrable health benefits of ITN use, especially among young children and pregnant women (Lengeler 2004; Gamble *et al.* 2007). The age group least likely to use ITNs are school-aged children (Noor *et al.* 2009b), and few data exist on patterns of net use and effectiveness of nets among this age group (Nevill *et al.* 1988; Luxemburger *et al.* 1994; Leenstra *et al.* 2003). In the absence of data from intervention studies, cross-sectional surveys can provide insight into the potential efficacy of ITNs. Surveys from Somalia (Noor *et al.* 2008) and Uganda (Pullan *et al.* 2010) found that sleeping under a net the previous night was associated with a 71% and 43% lower risk of *Plasmodium* infection in school-aged children. However, the potential protective efficacy of ITNs in reducing *Plasmodium* infection and anaemia among school children may not be the same in all settings owing to differences in the underlying intensity of malaria transmission and the relative contribution of other factors that contribute to anaemia in this age group, including undernutrition (Best *et al.* 2010) and helminth infections (Friedman *et al.* 2005; Koukounari *et al.* 2008; Smith & Brooker 2010).

We investigated putative risk factors, including reported net use, for *Plasmodium* infection and anaemia among school children in Kenya and explored how they vary across the different malaria ecologies that occur in the country. We analysed data from a recent nationwide school malaria survey in Kenya (Gitonga *et al.* 2010) and examined how the associations between reported net use, malaria parasitaemia and anaemia vary according to age and sex in the different malaria transmission settings.

## Methods

**Figure 1:**
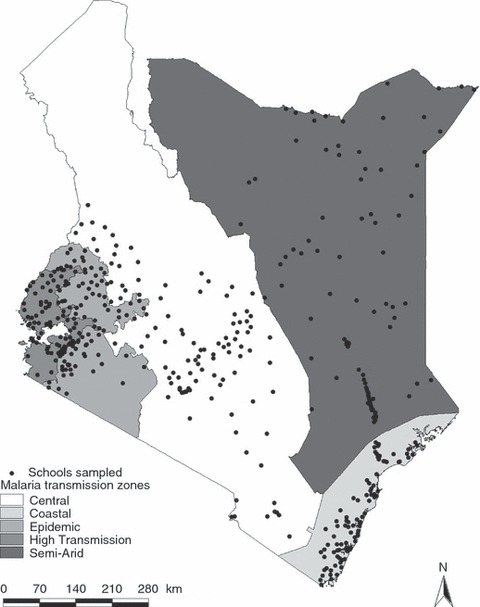
The geographical distribution of the 480 sampled schools by malaria transmission zones in Kenya, as based on a geostatistical model of *Plasmodium falciparum* prevalence (Noor *et al.* 2009a).

The survey design and procedures of the national survey conducted in 480 schools are detailed elsewhere (Gitonga *et al.* 2010). In brief, the surveys were conducted in two phases: the first phase involved 119 schools in coastal and north-eastern Kenya surveyed between September 2008 and March 2009 and the second phase comprised a sample of schools selected to allow for adequate spatial representation across the country (Figure 1), surveyed between May 2009 and March 2010. The selection of pupils in each school was the same for each survey phase: 11 boys and 11 girls were selected from classes 2 to 6 to achieve a desired sample of 110 children. In schools where the desired sample could not be achieved because of low enrolment, all the students in classes 2–6 were recruited.

### Survey procedures

Selected children were asked to provide a finger-prick blood sample which was used to assess *Plasmodium* infection in peripheral blood in all the 480 schools while anaemia was assessed in a randomly selected sub-sample of 399 schools. Children had both a rapid diagnostic test (RDT), which gave an on-the-spot diagnosis for malaria, and a thick and thin blood smear for subsequent microscopy. Different RDT types were used during the different survey phases. 72.8% of children were tested with either a ParaCheck-*Pf* device or a ParaCheck-*Pf* dipstick, while the rest were tested using OptiMAL-IT (17.3%) or CareStart Malaria P*f*/P*v* Combo (9.9%) RDTs. Blood slides were labelled and air-dried horizontally in a carrying case in the school and stained with 3% Giemsa for 45 min at the nearest health facility at the end of each day. All RDT-positive microscopy slides and a random sample of RDT-negative slides were examined by expert microscopists to ascertain *Plasmodium* infection. The RDT results were corrected using the microscopy results, and the slide-corrected RDT results were used as the definitive malaria diagnosis. Using the same finger-prick sample, haemoglobin concentration was assessed using a portable haemoglobinometer (HemoCue Ltd, Angelhölm, Sweden) and estimated to an accuracy of 1 g/l. A questionnaire was administered to children to obtain data on mosquito net ownership and use and whether the net was an ITN. Information was also collected on recent deworming, key socio-economic variables such as household construction and drinking water source. The children’s responses were entered electronically into ASUS Eee PC 1005P or Acer Aspire One d250 netbook computers using a customised Microsoft Access database and transmitted nightly to Nairobi through the mobile phone network. The geographical locations of schools were determined using a Garmin eTrex global positioning system (Garmin, Olathe, KS, USA).

### Ethical considerations

The study protocol received ethical approval from the Kenya Medical Research Institute and National Ethics Review Committee. Additional approval was provided by the appropriate national, provincial and district-level health and education authorities, who were briefed about the survey. At the school level, parental consent was based on passive, opt-out consent rather than written opt-in consent owing to the low risk and routine nature of the study procedures. Individual assent was obtained from each child before participation in the survey.

### Data analysis

Analysis was performed using Stata version 11.0 (Stata Corporation, College Station, TX, USA). *Plasmodium* infection and anaemia were assessed for their association with reported net use for each of the five malaria transmission zones. *Plasmodium* infection was defined on the basis of RDT results corrected with expert microscopy results. Anaemia was defined as a haemoglobin concentration <130 g/l for boys aged >15 years, <120 g/l for children aged 12–14 years and girls >15 years, <115 g/l for children aged 5–11 years and <110 g/l for children younger than 5 years, with adjustments made for the elevation of the children’s school (WHO 2001). Mosquito net use was defined as any child who reported having slept under a net the night before the survey. For the purposes of the current analysis, we assumed that all nets are treated nets. This is because the vast majority of nets used in Kenya today are treated nets (Noor *et al.* 2007; Hightower *et al.* 2010; Division of Malaria Control, Kenya National Bureau of Statistics & ICF Macro 2011). Furthermore, in practical terms, children are unlikely to be able to distinguish whether nets are treated or not.

The country is stratified according to malaria transmission intensity based on a geostatistical model that combines available data on *Plasmodium falciparum* infection prevalence and ecological and climate covariates in a Bayesian model-based geostatistical framework to predict the prevalence of infection across Kenya for the year 2009 (Noor *et al.* 2009a). This model identifies five malaria transmission zones: lakeside high; coastal; western highlands epidemic; central low-risk and semi-arid. Estimates of school-level prevalence of hookworm infection were derived from a geostatistical model of hookworm prevalence (Pullan *et al.* 2011), with prevalence stratified into low (0–21%) and high prevalence (>21) on the basis of the 90th percentile.

Prevalence estimates were estimated using random effects models to account for clustering occurring at the school level (Rabe-Hesketh & Skrondal 2008). Prevalence estimates of *Plasmodium* infection and anaemia in the proportion of children using nets were estimated using zero-inflated Poisson (ZIP) models to account for the excess of zero prevalence, while the proportion of children using nets was estimated using a multilevel mixed effects model. The ZIP model was favoured over the standard Poisson model on the basis of the Vuong test (Vuong 1989; Gitonga *et al.* 2010).

Univariable analysis of risk factors for *Plasmodium* infection and anaemia was undertaken within each transmission zone separately for each outcome using mixed effects logistic regression. To select candidate covariates for multivariable analysis, an inclusion criterion of *P* < 0.1 from a likelihood ratio test (LR test) was pre-specified after *a priori* inclusion of age, sex and net use. Covariates included mosquito net use, household wealth indicators such as household construction (floor and walls), availability of electricity and latrine access. In addition, data on altitude and location (whether urban or rural) of the school were entered into the malaria models while data on *Plasmodium* infection status, deworming history and school-level estimated hookworm prevalence were also entered into the anaemia models. Backward-stepwise selection of covariates was used to generate minimum adequate models. Excluded covariates (*P* > 0.1) were retested in the final models using LR tests to confirm lack of association; however, reported net use, age group and sex were retained as fixed terms in all models regardless of statistical significance because of their known importance.

After identifying covariates for inclusion in multivariate regression models within each transmission zone, three *a priori* interactions were investigated: (i) reported net use and sex, (ii) reported net use and age group in both models and (iii) age and sex in the anaemia model. The existence of heterogeneity in the odds ratios according to sex and age groups was assessed on the basis of LR tests in multivariable models, and interaction was included in the final model if *P* < 0.1. Stratum-specific odds ratios were derived from the final multivariable models.

## Results

A total of 49 975 children from 480 schools were included in the surveys, but only 43 285 (86.6%) had complete data on all covariates of interest and therefore included in the analysis for *Plasmodium* infection. Data on anaemia were collected from 41 884 children in 399 schools and 98% of these had complete data and were therefore included in the anaemia analysis. A similar number of boys (50.7%) and girls were included (Table 1), and the median age was 11 years (inter-quartile range, 10–13 years).

**Table 1:** The number of children examined and the percentage of primary school children in Kenya infected with *Plasmodium* spp. infection and anaemia are reported using an insecticide treated net (ITN) by strata. 95% binomial confidence intervals (CIs) are indicated in parenthesis

	*Plasmodium* infection (*n* = 43 285)*			Anaemia (*n* = 40 885)†		
		
	Number examined (%)	Prevalence of *Plasmodium* infection‡ (95% CI)	Proportion net use§ (95% CI)	Number examined (%)	Prevalence of anaemia‡ (95% CI)	Proportion net use§ (95% CI)
*Plasmodium* infection						
No	41 388 (95.6)	–	44.5 (42.4–46.6)	38 855 (95.0)	23.6 (22.1–25.1)	45.4 (43.2–47.7)
Yes	1897 (4.4)	–	34.8 (31.4–35.4)	2030 (5.0)	34.0 (30.7–37.4)	34.3 (31.1–37.6)
Anaemic						
No	–	–	–	31 025 (75.9)	–	45.6 (43.4–47.9)
Yes	–	–	–	9860 (24.1)	–	44.5 (42.0–46.9)
Reported net use						
No	24 150 (50.7)	5.2 (4.0–6.4)	–	22 448 (54.9)	22.4 (20.8–24.0)	–
Yes	19 135 (49.3)	3.4 (2.6–4.2)	–	18 437 (45.1)	26.2 (24.4–27.9)	–
Sex						
Male	21 925 (50.7)	4.5 (3.5–5.5)	42.5 (40.3–44.6)	20 735 (50.7)	26.0 (24.3–27.6)	43.2 (41.0–45.5)
Female	21 360 (49.3)	4.3 (3.3–5.2)	46.1 (43.8–48.3)	20 150 (49.3)	22.2 (20.7–23.7)	47.1 (44.7–49.4)
Age group						
5–9 years	10 610 (24.1)	4.5 (3.4–5.7)	48.9 (46.5–51.3)	9823 (24.0)	21.9 (20.1–23.8)	49.8 (47.4–52.3)
10–15 years	29 450 (68.0)	4.5 (3.5–5.5)	43.4 (41.2–45.6)	27 987 (68.5)	23.6 (22.1–25.1)	44.3 (42.1–46.6)
>15 years	3225 (7.5)	3.1 (2.0–4.1)	39.3 (36.5–42.2)	3075 (7.5)	36.0 (33.4–38.6)	40.3 (37.4–43.2)
Malaria transmission zone¶						
Lakeside high transmission	7361 (17.0)	17.6 (13.5–21.6)	33.3 (30.1–36.6)	7639 (18.7)	22.6 (19.6–25.5)	30.9 (27.9–33.9)
Coastal	9797 (22.6)	2.8 (2.0–3.7)	63.0 (59.7–66.4)	9626 (23.5)	39.2 (37.0–41.5)	63.0 (59.6–66.3)
Western highlands epidemic	10 578 (24.4)	2.3 (1.3–3.3)	35.5 (32.5–38.4)	8480 (20.7)	11.4 (10.1–12.8)	37.8 (34.4–41.1)
Central low-risk	10 879 (25.1)	0.5 (0.1–0.8)	38.7 (34.2–43.3)	10 477 (25.6)	13.4 (11.4–15.4)	40.1 (35.4–44.7)
Semi-arid north-eastern	4670 (10.8)	0.8 (0.3–1.4)	55.9 (48.8–63.0)	4663 (11.4)	42.6 (39.0–46.2)	55.9 (48.8–62.9)

* 6690 children excluded from the final analysis because of missing data.

† 999 children excluded from final analysis because of missing data.

‡ Prevalence and 95% CIs estimated using a zero-inflated Poisson model adjusting for clustering at the school level.

§ Proportion and 95% CIs estimated using a multilevel random effects model adjusting for clustering at the school level.

¶ Zones based on a geostatistical model of *Plasmodium* prevalence in Kenya (Noor *et al.* 2009a).

The overall microscopy-corrected RDT prevalence of *Plasmodium* infection was 4.4% [95% confidence interval (CI), 3.4–5.4%], and the prevalence of anaemia was 24.0% (95% CI, 22.5–25.5%). The prevalence of infection was highest in lakeside zone and lowest in central and semi-arid zones, whereas anaemia was highest in the coastal and semi-arid zones (Table 1). Overall, 44.9% (95% CI, 42.9–47.0%) of children reported having slept under a net the night before the survey; 42.5% of boys and 46.1% of girls reported using a net. Net use varied by transmission zone being highest in the coastal zone and lowest in the lakeside zone (Table 1).

### *Plasmodium* infection and its risk factors

**Figure 2:**
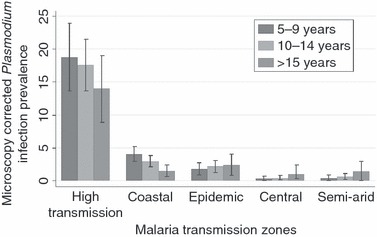
The prevalence of microscopy-corrected *Plasmodium* spp. infection in school children by age group across malaria transmission zones in Kenya, 2008–2010. Error bars indicate 95% binomial confidence intervals.

The importance of different risk factors was found to vary by malaria transmission zone (Tables 2 and 3). In particular, *Plasmodium* infection differed significantly by age group only in the lakeside and coastal zones, with lower risk with increasing age (Figure 2 and Table 3). In the multivariable analysis, girls had lower odds of infection in the lakeside zone and higher odds of infection in the coastal zone, but no association between sex and infection was found in other zones.

**Table 2:** Risk factors for *Plasmodium* infection among primary school children in Kenya stratified by malaria transmission zones, 2008–2010. Univariable odds ratios (OR) adjusted for clustering at the school level are shown with their corresponding 95% confidence intervals (95% CI)

	Lakeside high transmission (*n* = 7361)		Coastal (*n* = 9797)		Western highlands epidemic (*n* = 10 578)		Central low-risk (*n* = 10 879)		Semi-arid (*n* = 4670)	
					
	OR	*P*-value	OR	*P*-value	OR	*P*-value	OR	*P*-value	OR	*P*-value
Reported bed net use										
No *vs.* Yes	0.85 (0.73–0.99)	0.039	0.69 (0.54–0.90)	0.006	0.86 (0.62–1.18)	0.335	0.67 (0.32–1.34)	0.246	1.09 (0.55–2.16)	0.810
Sex										
Male *vs.* Female	0.80 (0.69–0.92)	0.002	1.35 (1.05–1.74)	0.018	0.79 (0.59–1.05)	0.101	0.85 (0.47–1.53)	0.585	1.28 (0.66–2.49)	0.458
Age group										
5–9 *vs.* 10–15 years	0.80 (0.68–0.95)		0.58 (0.42–0.79)		0.82 (0.58–1.15)		1.35 (0.67–2.71)		1.66 (0.56–4.93)	
5–9 *vs.* >15 years	0.52 (0.36–0.75)	0.001	0.21 (0.12–0.38)	<0.001	0.63 (0.34–1.14)	0.268	1.68 (0.53–5.31)	0.601	2.50 (0.71–8.87)	0.354
Wall type										
Bricks/cement *vs.* Mud/clay/other	1.57 (1.31–1.89)	<0.001	1.52 (1.03–2.25)	0.034	1.72 (1.00–2.96)	0.049	1.71 (0.68–4.30)	0.255	4.57 (0.60–34.98)	0.143
Floor										
Cement *vs.* Earth/wood/iron sheets	1.61 (1.34–1.92)	<0.001	1.24 (0.84–1.82)	0.275	2.09 (1.20–3.65)	0.009	1.15 (0.48–2.73)	0.751	3.41 (0.44–26.45)	0.240
Drinking water source										
Piped *vs.* Borehole/well	1.13 (0.86–1.48)		1.48 (0.94–2.31)		2.04 (1.00–4.16)		0.94 (0.32–2.71)			
Piped *vs.* Other*	1.15 (0.88–1.50)	0.589	1.56 (1.00–2.41)	0.131	1.89 (0.96–3.71)	0.140	1.24 (0.48–3.18)	0.824		
Electricity										
No *vs.* Yes	0.47 (0.31–0.72)	0.001	0.51 (0.22–1.18)	0.116	1.15 (0.51–2.60)	0.738	2.70 (0.91–8.00)	0.075		
Latrine										
No *vs.* Yes	0.90 (0.74–1.09)	0.293	0.56 (0.41–0.76)	<0.001	1.63 (0.70–3.78)	0.254	0.21 (0.09–0.51)	0.001	0.80 (0.38–1.75)	0.581
Urban										
No *vs.* Yes	0.54 (0.17–1.72)	0.296	2.76 (0.63–12.14)	0.178	3.5 (0.39–30.60)	0.264	12.62 (1.44–110.48)	0.022	0.28 (0.01–8.46)	0.465
Altitude										
0–1500 *vs.* >1500 m	0.48 (0.18–1.23)	0.126	Omitted†		0.05 (0.01–0.23)	<0.001	1.34 (0.27–6.67)	0.718	Omitted†	

* Other water sources included from neighbours, community water tanks and buying.

† Variables were omitted in the models because of collinearity.

**Table 3:** Risk factors for *Plasmodium* infection among primary school children in Kenya stratified by malaria transmission zones, 2008–2010. Multivariable odds ratios (OR) adjusted for clustering at the school level are shown with their corresponding 95% confidence intervals (95% CI)

	Lakeside high transmission (*n* = 7361)		Coastal (*n* = 9797)		Western highlands epidemic (*n* = 10 578)		Central low-risk (*n* = 10 879)		Semi-arid (*n* = 4670)	
					
	OR	*P*-value	OR	*P*-value	OR	*P*-value	OR	*P*-value	OR	*P*-value
Reported bed net use										
No *vs.* Yes	0.89 (0.76–1.05)	0.160	0.69 (0.53–0.90)	0.006	*		0.70 (0.34–1.45)	0.341	1.06 (0.53–2.13)	0.859
Sex										
Male *vs.* Female	0.77 (0.67–0.89)	<0.001	1.39 (1.08–1.79)	0.011	0.64 (0.45–0.91)†	0.014	0.86 (0.47–1.56)	0.610	1.35 (0.69–2.65)	0.378
Reported net use by sex										
Males										
Net non-users *vs.* Net users					0.65 (0.41–1.02)	0.062				
Females										
Net non-users *vs.* Net users	–		–		1.14 (0.74–1.78)	0.537	–		–	
Age group										
5–9 *vs.* 10–15 years	0.79 (0.67–0.93)		0.54 (0.40–0.75)		0.80 (0.56–1.13)		1.30 (0.64–2.64)		1.75 (0.58–5.21)	
5–9 *vs.* >15 years	0.48 (0.34–0.70)	<0.001	0.18 (0.10–0.33)	<0.001	0.58 (0.32–1.06)	0.146	1.64 (0.51–5.29)	0.659	2.68 (0.75–9.57)	0.296
Floor										
Cement *vs.* Earth/wood/iron sheets	1.52 (1.27–1.83)	<0.001	–		2.09 (2.00–3.65)	0.010	–		–	
Electricity										
No *vs.* Yes	0.59 (0.39–0.91)	0.017	–		–		3.09 (0.88–10.85)	0.078	–	
Latrine										
No *vs.* Yes	–		0.57 (0.42–0.78)	<0.001	–		0.15 (0.06–0.39)	<0.001	–	
Urban										
No *vs.* Yes	–		–		–		6.29 (1.03–38.37)	0.046	–	
Altitude										
0–1500 *vs.* >1500 m	–		–		0.05 (0.01–0.22)	<0.001	–		–	
Likelihood ratio test for interaction between										
Reported net use and sex		0.832		0.421		0.069		0.623		0.725
Reported net use and age group		0.887		0.145		0.250		1.000		0.167

–, Variables excluded from the final model.

* There was statistical evidence of an interaction; the stratum-specific results are therefore reported.

† Effect of sex on anaemia in non-net users.

*Plasmodium* infection and reported net use were significantly associated in the coastal zone with a 31% (95% CI, 10–47%) reduction in the odds of infection (*P* = 0.006). Although there was evidence in the univariable analysis of a 15% reduction in the odds of infection in children who reported using nets in the lakeside zone (Table 2), this effect was not apparent after adjusting for potential confounders (Table 3). The results from the LR tests in the multivariable models indicated that there was borderline variation in the association between infection and net use by sex in the western highlands epidemic zone, with a 35% reduction in the odds of infection among male net users. In the central and semi-arid zones, there was no evidence of an association between net use and infection.

Lower odds of infection were associated with attending a school situated at an elevation of >1500 m in the western highlands epidemic zone, whereas attending a school in an urban location was associated with higher odds of infection in the central zone. Finally, lower infection levels were associated with increased socio-economic status in all zones except in the semi-arid zone.

### Anaemia and its risk factors

As expected, the risk factors for anaemia varied according to malaria transmission zone (Table 4 and 5). In all zones, older children (>15 years) were associated with higher odds of anaemia while girls had lower odds of infection in the western highlands epidemic and semi-arid zones (Table 5). In the coastal, western highlands and semi-arid zones, there was evidence of an interaction between sex and age group (LR test *P* < 0.001), indicating no differences in the odds of anaemia in younger children (5–9 and 9–15 years) by sex while among children aged >15 years, girls had lower odds of infection.

**Table 4:** Risk factors for anaemia among primary school children in Kenya stratified by malaria transmission zones, 2008–2010. Univariable odds ratios (OR) adjusted for clustering at the school level are shown with their corresponding 95% confidence intervals (95% CI)

	Lakeside high transmission (*n* = 7639)		Coastal (*n* = 9626)		Western highlands epidemic (*n* = 8480)		Central low-risk (*n* = 10 477)		Semi-arid (*n* = 4663)	
					
	OR	*P*-value	OR	*P*-value	OR	*P*-value	OR	*P*-value	OR	*P*-value
Reported bed net use										
No *vs.* Yes	0.89 (0.78–1.01)	0.067	0.91 (0.83–1.00)	0.044	0.87 (0.75–1.01)	0.077	0.83 (0.73–0.95)	0.008	0.95 (0.83–1.09)	0.483
Sex										
Male *vs.* Female	0.94 (0.84–1.05)	0.248	0.72 (0.66–0.78)	<0.001	0.81 (0.71–0.93)	0.003	0.91 (0.80–1.02)	0.101	0.75 (0.66–0.84)	<0.001
Age group										
5–9 *vs.* 10–15 years	0.92 (0.81–1.05)		0.96 (0.85–1.08)		0.95 (0.80–1.12)		0.95 (0.83–1.08)		0.88 (0.75–1.02)	
5–9 *vs.* >15 years	1.30 (0.99–1.70)	0.020	1.27 (1.08–1.49)	<0.001	1.88 (1.45–2.44)	<0.001	1.74 (1.34–2.27)	<0.001	1.48 (1.17–1.86)	<0.001
*Plasmodium* infection										
No *vs.* Yes	1.54 (1.33–1.79)	<0.001	1.13 (0.87–1.46)	0.351	2.20 (1.57–3.09)	<0.001	3.02 (1.37–6.67)	0.006	1.05 (0.54–2.06)	0.876
Wall type										
Bricks/cement *vs.* Mud/clay/other	1.07 (0.92–1.23)	0.379	1.09 (0.96–1.21)	0.232	1.04 (0.83–1.29)	0.754	0.99 (0.86–1.14)	0.899	1.20 (0.99–1.47)	0.064
Floor										
Cement *vs.* Earth/wood/iron sheets	1.01 (0.88–1.16)	0.862	1.08 (0.95–1.21)	0.231	1.00 (0.82–1.22)	0.985	1.11 (0.96–1.29)	0.154	1.16 (0.95–1.41)	0.152
Drinking water source										
Piped *vs.* Borehole/well	1.03 (0.81–1.30)		1.08 (0.94–1.23)		0.96 (0.70–1.32)		1.20 (0.96–1.49)		1.09 (0.83–1.42)	
Piped *vs.* Other*	1.02 (0.82–1.28)	0.976	1.04 (0.92–1.19)	0.552	0.88 (0.67–1.17)	0.532	1.02 (0.84–1.25)	0.171	1.24 (0.96–1.61)	0.156
Electricity										
No *vs.* Yes	0.76 (0.55–1.05)	0.098	0.99 (0.82–1.21)	0.953	0.95 (0.61–1.49)	0.831	0.80 (0.63–1.02)	0.071	0.93 (0.65–1.33)	0.703
Latrine										
No *vs.* Yes	1.03 (0.88–1.19)	0.729	0.96 (0.88–1.07)	0.538	1.09 (0.82–1.44)	0.552	0.93 (0.77–1.11)	0.413	0.89 (0.77–1.02)	0.094
Dewormed in the last year										
No *vs.* Yes	0.88 (0.72–1.07)	0.200	0.85 (0.77–0.93)	<0.001	1.02 (0.86–1.20)	0.832	0.81 (0.70–0.94)	0.005	0.91 (0.79–1.04)	0.150
Estimated hookworm prevalence										
0–21% *vs.* >21%	1.30 (0.76–2.23)	0.338	1.09 (0.89–1.34)	0.384	0.80 (0.46–1.37)	0.414	Omitted†		Omitted†	

* Other water sources included from neighbours, community water tanks and buying.

† Variables were omitted in the models because of collinearity.

**Table 5:** Risk factors for anaemia among primary school children in Kenya stratified by malaria transmission zones, 2008–2010. Multivariable odds ratios (OR) adjusted for clustering at the school level are shown with their corresponding 95% confidence intervals (95% CI)

	Lakeside high transmission (*n* = 7639)		Coastal (*n* = 9626)		Western highlands epidemic (*n* = 8480)		Central low-risk (*n* = 10 477)		Semi-arid (*n* = 4663)	
					
	OR	*P*-value	OR	*P*-value	OR	*P*-value	OR	*P*-value	OR	*P*-value
Reported bed net use										
No *vs.* Yes	*		0.91 (0.83–1.00)	0.048	0.90 (0.77–1.04)	0.150	0.86 (0.75–0.98)	0.023	1.01 (0.88–1.16)	0.912
Sex										
Male *vs.* Female	0.89 (0.78–0.95)†	0.108	*		*		0.92 (0.82–1.03)	0.174	*	
Reported net use by sex										
Males										
Net non-users *vs.* Net users	0.79 (0.66–0.95)	0.012	–		–		–		–	
Females										
Net non-users *vs.* Net users	1.01 (0.85–1.20)	0.895	–		–		–		–	
Age group										
5–9 *vs.* 10–15 years	0.93 (0.81–1.06)		0.97 (0.82–1.14)‡	0.677	1.05 (0.82–1.34)‡	0.723	0.93 (0.81–1.06)	0.278	0.88 (0.71–1.10)‡	0.266
5–9 *vs.* >15 years	1.34 (1.02–1.75)	0.017	3.38 (2.66–4.29)§	<0.001	2.53 (1.82–3.51)§	<0.001	1.67 (1.28–2.18)	<0.001	1.69 (1.25–2.28)§	0.001
Sex by age group										
5–9 years										
Male *vs.* Female	–		0.92 (0.75–1.13)	0.428	1.05 (0.79–1.40)	0.735	–		0.83 (0.64–1.08)	0.157
10–15 years										
Male *vs.* Female	–		0.88 (0.80–0.98)	0.015	0.87 (0.73–1.03)	0.109	–		0.77 (0.67–0.90)	0.001
>15 years										
Male *vs.* Female	–		0.13 (0.10–0.17)	<0.001	0.36 (0.21–0.63)	<0.001	–		0.50 (0.34–0.74)	0.001
*Plasmodium* infection										
No *vs.* Yes	1.54 (1.33–1.79)	<0.001	–		2.24 (1.60–3.13)	<0.001	3.00 (1.35–6.66)	0.007	–	
Wall type										
Bricks/cement *vs.* Mud/clay/other	–		–		–		–		1.21 (0.99–1.47)	0.057
Dewormed in the last year										
No *vs.* Yes	–		0.86 (0.78–0.94)	0.001	–		0.83 (0.72–0.97)	0.017	–	
Likelihood ratio test for interaction between										
Net use and sex		0.051		0.270		0.113		0.669		0.274
Net use and age group		0.178		0.570		0.168		0.472		0.348
Sex and age group		0.181		<0.001		0.002		0.212		0.090

–, Variables excluded from the final model on the basis, *P*-value >0.1.

* There was statistical evidence of an interaction; the stratum-specific results are therefore reported.

† Effect of sex on anaemia in non-net users.

‡ Effect of age in males, age group 5–9 *vs.* 10–15 years.

§ Effect of age in males, age group 5–9 *vs.* >15 years.

*Plasmodium* infection was associated with higher odds of anaemia in the lakeside, western highlands epidemic and central zones. Reported net use was associated with lower odds of anaemia in coastal and central zones and among male net users in the lakeside zone; no association was evident in the western highlands epidemic and semi-arid zones (Table 5). Recent deworming was associated with lower odds of anaemia in coastal and central zones, with no evidence of an association in the other zones. There was statistical evidence of variation in the odds ratios for the association between reported ITN use and anaemia, by sex in the lakeside zone (LR test *P* = 0.051); however, there was no evidence of variation in the other zones or by age group.

## Discussion

To effectively target malaria control interventions, an understanding of the potential efficacy of interventions against malaria and related co-morbidities, such as anaemia, is necessary. To our knowledge, this study presents the first nationwide analysis of the association between reported net use, *Plasmodium* infection and anaemia in school children in a country with diverse malaria and nutritional ecologies. Results suggest that reported net use was associated with reduction in the odds of *Plasmodium* infection among all children in the coastal zone, and there was borderline evidence of a 35% reduction in the odds of infection among boys in the western highlands epidemic zone; no protective effect was observed in all other malaria transmission zones. Reported net use was associated with reduced odds of anaemia in the central and coastal zones and among the boys living in high lakeside zone.

Since the completion of the current work, the results of the 2010 Kenya Malaria Indicator Survey (MIS) have been published (Division of Malaria Control, Kenya, National Bureau of Statistics & ICF Macro 2011). Unlike in the 2007 MIS, this MIS assessed ITN use, malaria parasitaemia and anaemia among school-age children (5–14 years). There are, however, notable differences in the findings of the 2010 MIS and our school survey results, for example, in the 2010 MIS, 34.2% and 27.8% of children reported sleeping under any net or ITN, respectively, whereas these figures were 44.9% and 19.0%. The disparity may be explained by temporal changes in bed net ownership and use and the unreliability of school children reports on net treatment status. The 2010 MIS also reported a higher prevalence of infection: 13.3% based on microscopy compared to 4.4% in our school surveys. Such an increase may reflect temporal changes in transmission, which is consistent with recent studies that have shown a rise in infection prevalence in the lakeside high transmission zone (Zhou *et al.* 2011). An in-depth analysis on the congruence between household surveys, such as those in the 2010 MIS, and school surveys in malaria surveillance is the subject of ongoing work.

The observed lack of an association between net use and infection in the lakeside zone contrasts other studies that have reported a reduced risk of malaria infection among school-aged children who use nets (Baliraine *et al.* 2009; Fillinger *et al.* 2009). Possible factors for the lack of protective efficacy of nets in the lakeside zone include the high intrinsic intensity of transmission as well as infrequent net use and poor quality of nets being used. For example, studies in Kenya indicate that school-age children are most likely to sleep under poor quality nets (Githinji *et al.* 2010; Atieli *et al.* 2011) and household sleeping arrangements, such that school-aged children sleep on the floor and in areas where it is not possible to hang nets, which may affect the consistent use of nets by this age group (Alaii *et al.* 2003; Iwashita *et al.* 2010). In addition, this study was carried out 2–3 years after the last mass distribution of LLINs in Kenya in 2006, and as has been shown in other studies, (Ashton *et al.* 2011; Rehman *et al.* 2011) the physical quality of nets in use deteriorates quickly. In high transmission zones, maintaining high ITN coverage and use in conjunction with complementary malaria interventions are required to effectively reduce malaria transmission and disease burden (Smith *et al.* 2009). In 2008, indoor residual spraying was conducted in selected districts in the lakeside and western highlands zones to augment the effect of ITN distribution, while in 2011, mass distribution of LLINs aimed at universal coverage was conducted in the same districts. Within a school context, the use of intermittent preventive treatment (IPT) or intermittent screening and treatment (IST) may also help reduce the burden of malaria in high transmission settings (Brooker *et al.* 2008, 2010; Clarke *et al.* 2008; Barger *et al.* 2009; Brooker 2009; Nankabirwa *et al.* 2011). In central and semi-arid transmission zones, the cost-effectiveness of ensuring universal ITN coverage remains unclear because of low overall level of malaria transmission.

The effect of reported net use on anaemia is likely to be mediated by malaria transmission intensity and the presence of other aetiological factors for anaemia (Korenromp *et al.* 2004), including underlying nutritional factors (Best *et al.* 2010), prevalence and intensity of helminth infection (Kabatereine *et al.* 2007; Koukounari *et al.* 2008) and mix of helminth species (Midzi *et al.* 2008). Kenya is characterised by marked geographical diversity in such factors, including malaria (Noor *et al.* 2009a), hookworm infection (Pullan *et al.* 2011) and undernutrition (Kenya National Bureau of Statistics and ICF Macro 2010). In the lakeside and western highlands zones, where the prevalence of undernutrition is low and high prevalence of *Plasmodium* and helminth infections and co-infection can be seen (Brooker *et al.* 2012), integrated malaria and helminth control programmes are likely to be beneficial (Brooker *et al.* 2007). In the coastal zone, integrated malaria and helminth control with some form of school feeding and/or micronutrient supplementation is warranted (Halliday *et al.* 2012). Finally, in the central and semi-arid zones, it would seem that malaria and helminth control should be geographically targeted to only selected foci, but universal coverage of nutritional programmes would be beneficial.

Our results also provide useful insight into the geographical variation in the relative importance of other risk factors for malaria and anaemia. Notably, there are differences in the magnitude and direction of the effect of risk factors such as sex, age and socio-economic indicators. First, the observed differences in infection risk by age (Figure 2) are consistent with the exposure-related acquired immunity, with infection risk declining with age in high malaria transmission settings and a similar risk among age groups in low transmission settings. Second, the sex differences in infection risk by zone lend weight to the importance of exposure-related factors in explaining sex differences in infection risk, rather than some intrinsic differences in susceptibility to infection (Dunn *et al.* 2011). Third, observed differences in the relative importance of the socio-economic indicator variables may reflect the difficulties of a composite socio-economic indicator index in a country with heterogeneous communities.

This study is not without its limitations. First, net use and net treatment status were not directly ascertained. This may lead to misclassification of net users and non-users and ITNs and non-ITNs, thereby underestimating the effect size. However, a study in Uganda comparing school children’s reports on bed net ownership and community-based reports showed that school children can reliably report community-level bed net ownership (Ndyomugyenyi & Kroeger 2007). Furthermore, the misclassification of ITNs and non-ITNs may be less of a problem as LLIN coverage increases with the assumption that most nets being used will be LLINs. The results from the 2010 MIS indicate that about 80% of nets in use are treated nets and therefore the misclassification of nets is unlikely to explain the observed results. Second, this study was conducted 2–3 years after the last mass distribution of nets in Kenya, and the quality of bed nets was not assessed; therefore, the observed protective effectiveness in our study could be an underestimation. Finally, the study utilises data from a cross-sectional survey and is therefore subject to the caveats regarding inference and causality (Rothman *et al.* 2008).

In conclusion, our results demonstrate that the use of mosquito nets by school children varies markedly across Kenya, and importantly, their protective effects for *Plasmodium* infection and anaemia vary according to malaria transmission zones. The results also identify differences by zones in other risk factors associated with infection and anaemia. The study highlights the need for scaling up ITN coverage in the lakeside high transmission and western highlands epidemic regions and that ITNs alone are unlikely to control malaria in such settings, and there is a need to implement a suite of malaria interventions, including IRS and IPT. A geographically targeted, integrated approach for tackling anaemia in the country is also warranted.
